# Editorial: Advances in metabolism and chemodiversity – focus – anthocyanin and proanthocyanin: biosynthesis, accumulation, regulation

**DOI:** 10.3389/fpls.2023.1222082

**Published:** 2023-08-16

**Authors:** Md Abdur Rahim, Prashant Misra, James R. Ketudat Cairns

**Affiliations:** ^1^ Department of Genetics and Plant Breeding, Sher-e-Bangla Agricultural University, Dhaka, Bangladesh; ^2^ Plant Sciences and Agrotechnology Division, Indian Institute of Integrative Medicine (CSIR), Jammu, India; ^3^ Center for Biomolecular Structure, Function and Application & School of Chemistry, Institute of Science, Suranaree University of Technology, Nakhon Ratchasima, Thailand

**Keywords:** plant pigments, transcriptomics, metabolomics, transcription factors, structural genes

Anthocyanins and proanthocyanidins are water soluble, bioactive, secondary metabolites of the flavonoid class of plant pigments. They are responsible for red, purple, blue colours of vegetative and reproductive parts of plants (**Figure 1**) while proanthocyanidins are brown colour in some tissues (seed, peel and bark) or nonvisible pigments (Rahim et al., 2014; Zhao et al., 2010; Xia et al., 2022). They improve the fruit and vegetable quality and are beneficial to human health. In addition, they have been implicated in diverse aspects of plant biology, particularly in tolerance/resistance against abiotic and biotic stresses.

**Figure 1:**
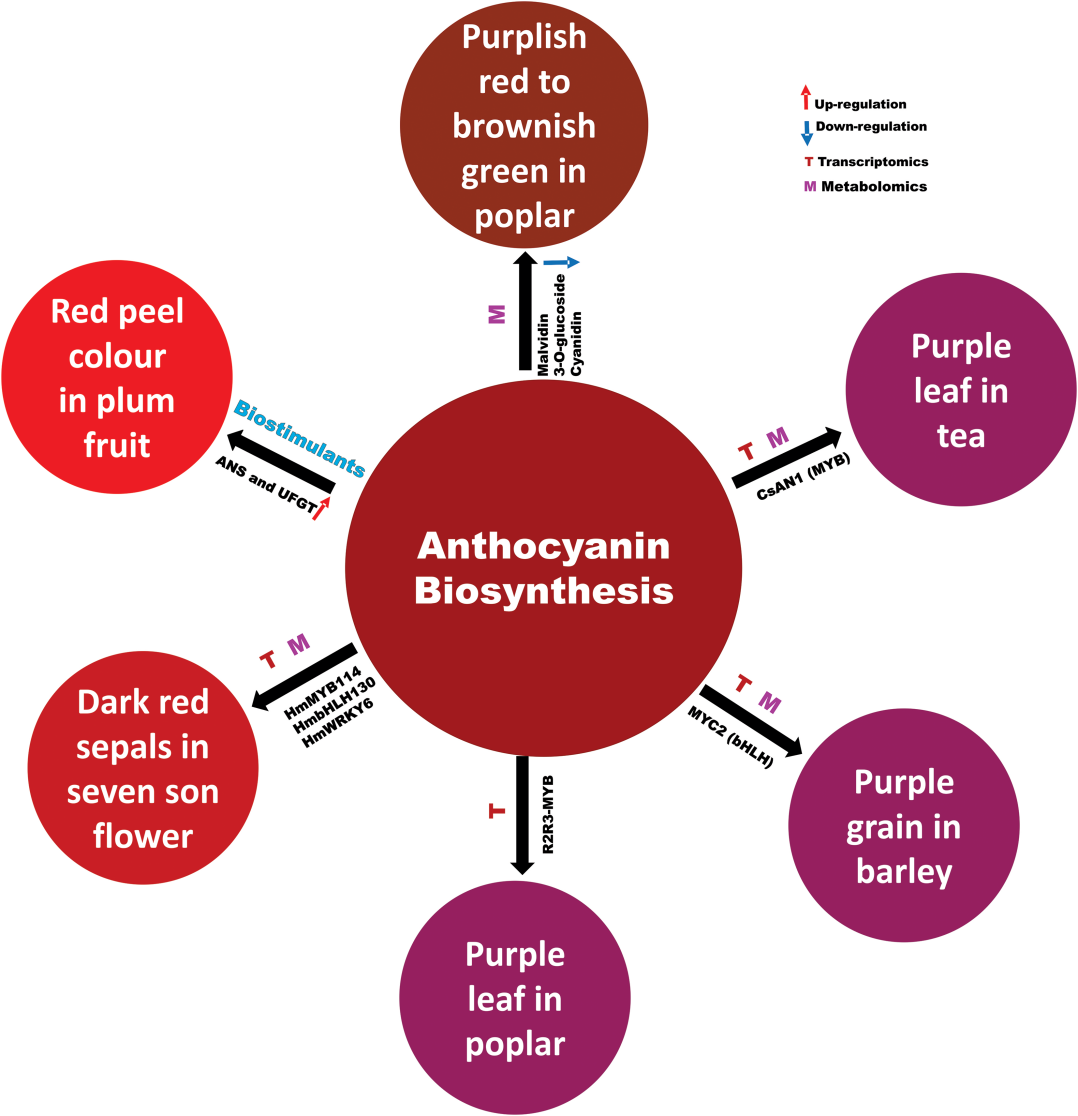
Regulation of anthocyanin biosynthesis in different plants.

The late steps in the flavonoid biosynthesis catalysed by the enzymes *dihydroflavonol reductase* (*DFR*) and *anthocyanidin synthase* (*ANS*) define the committed steps involved in anthocyanin and proanthocyanidin biosynthesis. The structural genes, including *DFR* and *ANS* are co-ordinately regulated by a complex of myeloblastosis (MYB), basic helix-loop-helix (bHLH) and WD40-repeat proteins (MBW), which is conserved across diverse plant species. Transcription factors (TFs), belonging to other families, along with MBW complex fine tune the anthocyanin biosynthesis under different exogenous and endogenous cues (Naik et al., 2022). Most of the available information on the molecular basis of anthocyanin biosynthesis and its regulation has evolved from studies on *A. thaliana*, maize, apple, and petunia (Naik et al., 2022). However, owing to the importance of these molecules in plants, it is imperative to study diverse aspects of anthocyanin biology in other economically important plants.

Tremendous progress has been made towards understanding, characterising, engineering, regulating, and medical application of these pigments over the past decades. This Research Topic contains six original research articles focusing on anthocyanin biosynthesis and its accumulation in different plant parts, including leaves, flowers, grains and fruits.

Purple tea leaves have gained popularity due to the health benefits of the anthocyanins that they contain. Previously, Sun et al. (2016) showed that the red-purple colour in the young leaves of Zijuan tea was directly related to the expression of the MYB TF family anthocyanin 1 (AN1) and bHLH and WD40 proteins that form a MBW complex to upregulate anthocyanin synthesis gene transcription. Huang et al. have extended that work with targeted metabolomics and transcriptomics to show that expression of the *anthocyanidin synthase* gene *ANS1* is highly correlated with anthocyanins and *AN1* expression in crosses of Zijuan with the green tea Fudingdabaicha. They further identified a 160 bp deletion in the *AN1* gene that can explain the lack of *AN1* function and the subsequent green phenotype of Fudingdabaicha tea.

Targeted metabolomics and transcriptomics were also used by Li et al. to study the origin of the bright red sepals that develop in the seven-son flower tree (*Heptacodium miconioides*) in autumn. They identified cyanidin glycosides as the pigments that increased in correlation with sepal colour. Moreover, they correlated gene expression of anthocyanin synthesis pathway enzymes with flower colour, and anthocyanin accumulation and with the expression of MYB, bHLH, WRKY, and NAC TF genes may control their transcription. Several of these TFs could drive gene expression from the *HmCHS4* (*chalcone synthase*) and *HmDFR1* (*dehydroflavone reductase*) gene promoters when transiently expressed in tobacco leaves. This identification of the anthocyanins, biosynthetic enzymes and regulatory factors involved in sepal colour development may help to guide development of ornamental flowers with pigmented sepals.

Chen et al. described the mechanism of the transition of leaf colour in *Populus* × *euramericana* ‘Zhonghuahongye’ at three different stages of leaf development through metabolic analysis. Ten core metabolites that were mostly flavonoids revealed substantial differences in all comparisons. The transition of red leaf colour from a bright-purplish red to a brownish green was due to the downregulation of flavonoid metabolites, including malvidin 3-O-glucoside and cyanidin, which provides a genetic basis for the improvement of poplar cultivars.

In another integrated transcriptome and metabolome study, Peng et al. analysed the molecular basis of anthocyanin biosynthesis regulation in different popular cultivars displaying varying contents of anthocyanin in their leaves. The authors identified 39 anthocyanin compounds, which showed differential profiles in the leaves of these different popular cultivars. Transcriptome analysis revealed several differentially regulated genes in the selected poplar cultivars. The expression patterns of certain differentially regulated genes correlated with the anthocyanin metabolites. Based on the correlation analysis, the authors identified one R2R3-MYB SG5 sub-group transcription factor gene, *Podel.04G021100*. The SG5 sub-group of R2R3-MYB TF regulates structural genes involved in the biosynthesis of flavonoids (Schwinn et al., 2016). Thus, the close correlation of its expression with several anthocyanin metabolites and its phylogenetic positioning imply that *Podel.04G021100* might be involved in regulation of anthocyanin biosynthesis in poplar, although functional studies are required to ascertain its function.

Likewise, Xu et al. studied the molecular basis of differential anthocyanin pigmentation in grains of three Tibetan hulless barley (qingke) varieties displaying contrasting grain pigmentation using an integrated transcriptomics and metabolomics approach. Metabolomic analysis at different developmental stages revealed differential accumulation of flavonoids including anthocyanin in the grains of different varieties. Transcriptome analysis provided information about several differentially regulated genes in the grains of three varieties. Integration of metabolomics and transcriptomics data revealed several structural genes of the flavonoid biosynthesis including genes that encode modifying enzymes (*glycosyltransferases*, *methyltransferase* and *malonyltranferase*) of anthocyanidins, which may account for variation in the quality and quantity of the anthocyanin pigmentation. A transcription factor gene *HvMYC2* was found to be co-expressed with *HvF3H*, *HvGT1*, *HvGT2*, and *HvMAT*, suggesting that it might be involved in the regulation of anthocyanin biosynthesis and modifying genes. Promoter transactivation assay and yeast 1 hybrid system confirmed that HvMYC2 can transactivate the expression of *HvF3H*. Further, the study demonstrates that HvMYC2 might play a role in mediating UV-B and low temperature induced anthocyanin pigmentation.

Effects of biostimulants on the accumulation of carbohydrates and anthocyanin biosynthesis in ‘Yinhongli’ plum are described by Yao et al., who treated the developing plum fruit surface with humic acid, seaweed extract and amino acids to elucidate their influence on carbohydrates and anthocyanin biosynthesis during ripening. All biostimulants potentially enhanced the total soluble solids (TSS) and titratable acidity (TA), as well as anthocyanin accumulation in the red skinned plum (‘Yinhongli’) compared to green skinned plum. Moreover, the amino acids treatment significantly increased the expression of anthocyanin biosynthetic genes, particularly *CHS* and *flavonoid 3-O-glucosyltransferase* (*UFGT*), leading to early pigment accumulation compared to the control. This study also showed a significant correlation between total sugar and anthocyanin content during fruit ripening and provides an avenue for further investigation of the interactions between anthocyanin biosynthetic and sugar metabolic pathways in plum.

These studies highlight the utility of integrated approaches involving transcriptomics and metabolomics for the identification of candidate regulators of anthocyanin biosynthesis and provide basic knowledge for anthocyanin production for health and ornamental applications. Understanding the molecular basis of the anthocyanin biosynthesis will be helpful in improving the content of these health promoting molecules in fruits and vegetables. However, in order to explore the full potential of health beneficial effects of anthocyanin, it is imperative to address bioavailability and stability issues associated with anthocyanins. These limitations might be tackled by approaches like their complexation with lipids and proteins, and nanoencapsulation (Shen et al., 2022). However, such modifications are not practical in case of anthocyanin taken from natural sources like fruit and vegetables. Therefore, future research should also be focussed on the possibilities of engineering of source plants to modify the anthocyanins for improving their bioavailability.

## Author contributions

All authors listed have made a substantial, direct, and intellectual contribution to the work and approved it for publication.
